# Vertical Jumping Tests *versus* Wingate Anaerobic Test in Female Volleyball Players: The Role of Age

**DOI:** 10.3390/sports4010009

**Published:** 2016-02-05

**Authors:** Pantelis Theodoros Nikolaidis, Jose Afonso, Vicente Javier Clemente-Suarez, Jose Rafael Padilla Alvarado, Tarak Driss, Beat Knechtle, Gema Torres-Luque

**Affiliations:** 1 Department of Physical and Cultural Education, Hellenic Army Academy, Athens 16673, Greece; 2 Exercise Physiology Laboratory, Nikaia 18450, Greece; 3 Faculty of Sport, University of Porto, Porto 4200-450, Portugal; jafonsovolei@hotmail.com; 4 Department of Sport Science, European University of Madrid, Madrid 28670, Spain; vctxente@yahoo.es; 5 Department of Physical Education, Universidad de Los Llanos Occedentales Ezequiel Zamora, Barinitas 7001, Venezuela; joserafael.pa@gmail.com; 6 UFR STAPS, CeRSM (EA 2931), Université Paris Ouest Nanterre La Défense, Nanterre 92000, France; tarak.driss@u-paris10.fr; 7 Instutute of Primary Care, University of Zurich, Zurich CH-8006, Switzerland; beat.knechtle@hispeed.ch; 8 Area of Corporal Express, Faculty of Humanities and Science Education, University of Jaen, Jaen 3109, Spain; gtluque@ujaen.es

**Keywords:** age groups, anaerobic power, exercise mode, performance analysis, physiological evaluation, short-term power, team sport

## Abstract

Single and continuous vertical jumping tests, as well as the Wingate anaerobic test (WAnT), are commonly used to assess the short-term muscle power of female volleyball players; however, the relationship among these tests has not been studied adequately. Thus, the aim of the present study was to examine the relationship of single and continuous vertical jumps with the WAnT in female volleyball players. Seventy adolescent (age 16.0 ± 1.0 years, body mass 62.5 ± 7.1 kg, height 170.4 ± 6.1 cm, body fat 24.2% ± 4.3%) and 108 adult female volleyball players (age 24.8 ± 5.2 years, body mass 66.5 ± 8.7 kg, height 173.2 ± 7.4 cm, body fat 22.0% ± 5.1%) performed the squat jump (SJ), countermovement jump (CMJ), Abalakov jump (AJ), 30 s Bosco test and WAnT (peak power, P_peak_; mean power, P_mean_). Mean power in the Bosco test was correlated (low to large magnitude) with P_mean_ of the WAnT (*r* = 0.27, *p* = 0.030 in adolescents *versus* *r* = 0.56, *p* < 0.001 in adults). SJ, CMJ and AJ also correlated with P_peak_ (0.28 ≤ *r* ≤ 0.46 in adolescents *versus* 0.58 ≤ *r* ≤ 0.61 in adults) and with P_mean_ (0.43 ≤ *r* ≤ 0.51 *versus* 0.67 ≤ *r* ≤ 0.71, respectively) of the WAnT (*p* < 0.05). In summary, the impact of the Bosco test and WAnT on muscle power varied, especially in the younger age group. Single jumping tests had larger correlations with WAnT in adults than in adolescent volleyball players. These findings should be taken into account by volleyball coaches and fitness trainers during the assessment of short-term muscle power of their athletes.

## 1. Introduction

Volleyball is an intermittent high intensity team sport, where a combination of physical characteristics and aerobic and anaerobic capacity is necessary in order to perform a sequence of well-coordinated, high demand activities [1]. While a minimal level of aerobic capacity is necessary to cope with the demands of training and the game, maximal effort during short periods of time (e.g., jumping, hitting the ball) usually determines the outcome of a game [2]. Therefore, volleyball players should posses adequate short-term muscle power to compete at a high level [3,4]. Short-term muscle power is usually assessed using cycling—e.g., the 30 s Wingate anaerobic test, (WAnT) and jumping tests including single jumps such as the squat (SJ), countermovement (CMJ) and Abalakov jump (AJ), and continuous jumps such as the 30 s Bosco test—within a laboratory setting [5,6].

Although both jumping and cycling tests have been used extensively to assess short-term power in volleyball players, very few studies have been conducted to evaluate the relationship among these tests [7,8,9,10]. A study on young basketball players had shown large correlations between CMJ and two indices of the WAnT (peak power, P_peak_, and mean power, P_mean_) [7]. More recently, research on male volleyball players found a large correlation between P_mean_ of the WAnT with P_mean_ of the Bosco test [8], whereas large to very large correlations were observed among SJ, CMJ, P_peak_ and P_mean_ of the WAnT in young track-and-field athletes [9]. The sample size in the abovementioned studies was between 9 and 24.

The abovementioned studies have enhanced our understanding of the relationship of jumping tests with the WAnT; however, none of them has addressed the effect of chronological age on this relationship. The knowledge of the relationship between cycling and jumping tests would contribute to an optimal measurement and evaluation of short-term muscle power [11,12], which would be of great practical value for volleyball coaches and fitness trainers. Moreover, understanding the effect of chronological age on this relationship might help volleyball professionals to use these tests according to age. Additionally, sex-related physiological differences in performance and in game demands have been identified in volleyball [13,14], thereby rendering generalization of studies on male samples to female samples a risky venture. Therefore, the aim of the present study was to examine the relationship between jumping tests of short-term muscle power and the WAnT in a wide range of chronological ages of female volleyball players.

## 2. Methods

### 2.1. Participants

A cross-sectional design was used to examine the relationship of single and continuous jumping tests with the WAnT in female volleyball. To accomplish this aim, 178 female volleyball players (<18 years, *n* = 70; ≥18 years, *n* = 108) from teams in the region of Athens were measured in the context of their yearly routine physical fitness assessment (Table 1). Testing procedures were carried out on September 2014 during the preparative period of seasons 2014–2015, respectively. The participants were familiar with testing procedures, because the fitness battery was routinely administered to these teams in the past, *i.e.*, all participants have performed the particular fitness battery at least one time previously. They visited the laboratory where they were examined for anthropometric characteristics, single and continuous jumping tests and the WAnT under standard environmental conditions (temperature 22–24 °C and humidity 50%–54%). The study was carried out according to the ethical standards of Declaration of Helsinki of the World Medical Association in 1964 as it was modified in 2013 and approved by the local institutional review board. Informed consent was given by all players or their guardians (in the case of underage participants).

**Table 1:** Anthropometric characteristics of volleyball players according to age group.

Variable	Total (*n* = 178)	<18 Years (*n* = 70)	>18 Years (*n* = 108)	Mean Difference (95% CI)
Age (years)	21.1 ± 5.9	16.0 ± 1.0	24.8 ± 5.2 ^†^	−8.8 (−10.1; −7.6), *d* = −2.4
Body mass (kg)	64.9 ± 8.4	62.5 ± 7.1	66.5 ± 8.8 *	−3.9 (−6.4; −1.5), *d* = −0.5
Height (cm)	172.1 ± 7.0	170.4 ± 6.1	173.2 ± 7.4 *	−2.8 (−4.9; −0.7), *d* = −0.4
BMI (kg·m^−2^)	21.9 ± 2.2	21.5 ± 1.9	22.1 ± 2.4	−0.6 (−1.3; 0.1), *d* = −0.3
BF (%)	22.9 ± 4.9	24.2 ± 4.3	22.0 ± 5.1 *	2.2 (0.8; 3.7), *d* = 0.5

BMI = body mass index, BF = body fat percentage, CI = confidence intervals, *d* = effect size Cohen’s *d*; * and ^†^ significant difference at *p* < 0.01 and *p* < 0.001, respectively.

### 2.2. Protocols and Equipments

#### 2.2.1. Anthropometry

We used an electronic body mass scale (HD-351 Tanita, Arlington Heights, IL, USA) and a portable stadiometer (SECA, Leicester, UK) to measure body mass in the nearest 0.1 kg and stature in the nearest 1 mm with participants being barefoot and in minimal clothing, respectively. These measurements were used to calculate body mass index (BMI) as the quotient of body mass (kg) to stature squared (m^2^). Body fat percentage (BF) was calculated from the sum of 10 skinfolds [15], which were taken with a skinfold caliper (Harpenden, West Sussex, UK). Chronological age for each participant was calculated using a table of decimals of year [16].

#### 2.2.2. Single Jumping Tests and Bosco Test

The participants performed two trials for each jumping exercise (squat jump (SJ), countermovement jump without arm-swing (CMJ) and with arm-swing (Abalakov jump, AJ)) and the best result was recorded [17]. Height of each jump was estimated using the Opto-jump (Microgate Engineering, Bolzano, Italy) and was expressed in cm. The coefficient of variation of these single jumps has been reported previously as ~2%–3%, respectively [18,19]. The validity of the Opto-jump has been tested against force plate and has been shown to be very high (intraclass correlation coefficient (0.997–0.998)); however, the Opto-jump consistently provided ~1 cm higher score than force plate [20]. This discrepancy should be attributed to the fact that the participant did not assume the same body configuration during takeoff and landing [21]. The reliability of this system has been examined previously using intraclass correlation coefficient and coefficient of variation which indicated excellent reliability (~0.985 and ~2.7%, respectively) [20]. The Opto-jump system calculates jump height from jump’s flight time [21]. The Bosco test was conducted on the same equipment as the abovementioned jump tests. The participants were instructed to jump as high as possible for 30 s, while trying to retain short ground contact times [22]. They were also requested to keep their hands on their waist throughout the test. The mean power during the 30 s test was expressed in W·kg^−1^.

#### 2.2.3. Wingate Anaerobic Test

The WAnT was performed on a cycle ergometer (Ergomedics 874, Monark, Sweden) [23]. This ergometer was modified with an extra photocell, placed on the floor exactly under the lowest position of right pedal, which was connected to a PC so each revolution was recorded in order to calculate the main indices of the test. Briefly, participants were asked to pedal as fast as possible for 30 s against a braking force that was determined by the product of body mass in kg by 0.075. Peak power (P_peak_) was estimated as the average power over a 5 s period with the highest performance, which occurs usually in the first 5 s of the test. Mean power (P_mean_) was calculated as the average power during the 30 s period. Both P_peak_ and P_mean_ were expressed in W and W·kg^−1^. Fatigue index (FI) was calculated as FI = 100 × (P_peak_—minimal power)/P_peak_, where minimal power was the lowest power output over a 5 s period, occurring usually towards the end of the test. The coefficient of variation of power indices has shown ~1%–2% [19].

### 2.3. Statistical Analysis

Statistical analyses were performed using IBM SPSS v.20.0 (SPSS, Chicago, IL, USA). Data were expressed as mean and standard deviations of the mean (SD). An independent t-test examined differences between adolescent and adult participants. Mean difference together with 95% confidence intervals (CI) was calculated. To interpret the effect size for statistical differences in the t-test we used Cohen’s *d* classified as *d* ≤ 0.2, trivial; 0.2 < *d* ≤ 0.6, small; 0.6 < *d* ≤ 1.2, moderate; 1.2 < *d* ≤ 2.0, large; and *d* > 2.0, very large [24]. Pearson correlation coefficient r examined the relationships among jumping tests and the WAnT. To interpret the magnitude of correlations the following criteria were adopted: *r* ≤ 0.1, trivial; 0.1 < *r* ≤ 0.3, small; 0.3 < *r* ≤ 0.5, moderate; 0.5 < *r* ≤ 0.7, large; 0.7 < *r* ≤ 0.9, very large; and *r* > 0.9, almost perfect [25]. The level of significance was set at α = 0.05.

## 3. Results

The anthropometric characteristics of participants can be found in Table 1. Differences among age groups were shown for all variables, except BMI which approached but did not quite achieve statistical significance (*p* = 0.075). The younger age group was lighter and shorter with higher BF than the older groups (*p* < 0.05). These differences had a small magnitude.

The results of the jumping tests and WAnT can be found in Table 2. The age groups differed for all single jump tests (SJ, CMJ and AJ) and all indices of the WAnT (absolute and relative P_peak_ and P_mean_) (*p* < 0.05), except FI. In these tests, adults scored higher than adolescent volleyball players. These differences were of moderate magnitude for SJ, absolute P_peak_ and P_mean_, and small for CMJ, AJ, relative P_peak_ and P_mean_.

**Table 2:** Short-term muscle power of volleyball players according to age group.

Variable	Total (*n* = 178)	<18 Years (*n* = 70)	>18 Years (*n* = 108)	Mean Difference (95% CI)
SJ (cm)	23.6 ± 4.1	22.1 ± 3.1	24.6 ± 4.4 ^‡^	−2.5 (−4.0; −1.1), *d* = −0.7
CMJ (cm)	25.2 ± 4.4	24.0 ± 2.9	25.9 ± 5.1 *	−1.9 (−3.5; −0.3), *d* = −0.5
AJ (cm)	30.5 ± 4.9	29.4 ± 4.5	31.1 ± 5.1 *	−1.7 (−3.2; 0.3), *d* = −0.4
Bosco (W·kg^−1^)	25.8 ± 5.0	25.4 ± 4.6	26.0 ± 5.3	−0.6 (−2.2; 0.9), *d* = −0.1
P_peak_ (W)	574 ± 88	537 ± 75	598 ± 88 ^‡^	−61 (−86; −36), *d* = −0.7
P_peak_ (W·kg^−1^)	8.90 ± 0.95	8.63 ± 0.85	9.07 ± 0.97 ^†^	−0.45 (−0.73; −0.16), *d* = −0.5
P_mean_ (W)	423 ± 66	393 ± 52	444 ± 66 ^‡^	−51 (−70; −33), *d* = −0.9
P_mean_ (W·kg^−1^)	6.57 ± 0.81	6.33 ± 0.72	6.73 ± 0.82 ^‡^	−0.40 (−0.64; −0.16), *d* = −0.5
FI (%)	45.5 ± 7.5	45.7 ± 7.7	45.4 ± 7.5	0.4 (−2.0; 2.7), *d* < 0.1

SJ = squat jump, CMJ = countermovement jump, Abalakov = jump, P_peak_ = peak power, P_mean_ = mean power, FI = fatigue index during the Wingate anaerobic test (WAnT). The symbols *, ^†^ and ^‡^ denoted significance at *p* < 0.05, *p* < 0.01 and *p* < 0.001, respectively.

**Figure 1:**
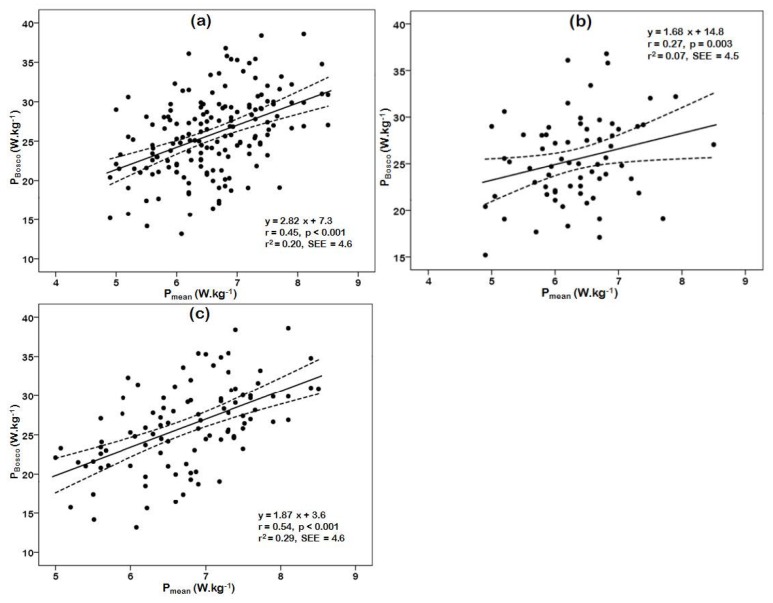
Relationship between mean power in the Wingate anaerobic test and mean power in the Bosco test in the total sample (**a**), adolescents (**b**), and adults (**c**). X axis represents mean power (W·kg^−1^) in Wingate anaerobic test; y axis represents mean power (W·kg^−1^) in Bosco test; SEE = standard error of estimate.

Mean power in the Bosco test was correlated (low to large magnitude) with relative P_mean_ in the WAnT (Table 3, Figure 1). SJ, CMJ and AJ were also correlated with P_peak_ (0.28 ≤ *r* ≤ 0.46 in adolescents *versus* 0.58 ≤ *r* ≤ 0.61 in adults) and with P_mean_ (0.43 ≤ *r* ≤ 0.51 *versus* 0.67 ≤ *r* ≤ 0.71, respectively) of the WAnT (*p* < 0.05).

**Table 3:** Correlations among cycling and jumping tests of short-term muscle power in volleyball players.

Variable	SJ			CMJ			AJ			Bosco Test		
	<18 Years	>18 Years	Total	<18 Years	>18 Years	Total	<18 Years	>18 Years	Total	<18 Years	>18 Years	Total
P_peak_ (W)	0.14	0.26 *	0.32 ^‡^	<0.01	0.25 *	0.26 ^‡^	0.17	0.16	0.22 ^†^	0.04	0.05	0.06
P_peak_ (W·kg^−1^)	0.33 *	0.61 ^‡^	0.58 ^‡^	0.28	0.58 ^‡^	0.54 ^‡^	0.46 ^‡^	0.59	0.56 ^‡^	0.08	0.46 ^‡^	0.33 ^‡^
P_mean_ (W)	0.28	0.41 ^‡^	0.45 ^‡^	0.15	0.39 ^†^	0.39 ^‡^	0.26 *	0.28 ^†^	0.32 ^‡^	0.21	0.14	0.18 *
P_mean_ (W·kg^−1^)	0.48 ^†^	0.71 ^‡^	0.67 ^‡^	0.43 ^†^	0.67 ^‡^	0.62 ^‡^	0.51 ^‡^	0.65 ^‡^	0.62 ^‡^	0.27 *	0.54 ^‡^	0.45 ^‡^
FI (%)	–0.23	0.02	–0.03	–0.23	0.02	–0.03	–0.06	0.05	0.01	–0.23	–0.01	–0.10

P_peak_ = peak power, P_mean_ = mean power, FI = fatigue index during the Wingate anaerobic test (WAnT), SJ = squat jump, CMJ = countermovement jump, AJ = Abalakov jump. The symbols *, ^†^ and ^‡^ denoted significance at *p* < 0.05, *p* < 0.01 and *p* < 0.001, respectively.

## 4. Discussion

The main findings of the present study were that (a) mean power in the Bosco test was correlated (low to large magnitude) with P_mean_ in the WAnT; (b) single jumps (SJ, CMJ and AJ) were correlated with P_peak_ and with P_mean_ of the WAnT; and (c) an age effect on the relationship between performance in jumping and cycling tests was observed (correlations of larger magnitude were found in adult rather than in adolescent volleyball players).

Although one would expect to observe a similar performance in the Bosco test and the WAnT, because both tests lasted the same time and required maximal effort, the findings confirmed this hypothesis only partially (*i.e.*, just in the case of adults). The magnitude of their correlation varied from small (adolescents) to large (adults), but even in the case of adults (Figure 1c), a large portion of variance (~71%) in performance in the Bosco test could not be accounted for by variance in performance in the WAnT. The differences in this correlation between adolescent and adult groups might be related to the athletes’ experience in the realization of these tests. Although the participants were not asked about the exact number of times they previously participated in the fitness battery, according to their higher athletic experience, it was assumed that the adult volleyball players have performed both tests on more occasions than the adolescents. On the contrary, the lower correlation in the adolescents might be due to them having less expertise in the coordination of their arms (e.g., in AJ) or in the eccentric phase of movement (e.g., in CMJ). An interpretation of this relationship between the Bosco test and the WAnT might be the different mode of exercise (jumping *versus* cycling), which recruited different muscle groups, and the role of body mass (it moved against gravity in the former mode, whereas it was supported by the saddle in the latter mode).

The lower correlation between the Bosco test and the WAnT in the adolescent volleyball players compared to their older counterparts indicated a lower ability to “translate” anaerobic power as measured in the cycling test into a continuous jumping performance for the former group. This variation might result from the effect of a long-term training process, including practice in jumping during training and game-play in the latter group, which allowed them to perform continuous jumping more efficiently. Similarly, SJ, CMJ and AJ correlated with P_peak_ and with P_mean_ of the WAnT with higher magnitude in the adult compared to the adolescent volleyball players. Furthermore, it was likely that the lower correlations in the adolescent participants might be due to the choice of the braking force during the WAnT [26,27,28,29].

Even if the findings of the present study suggested that the 30 s Bosco test should not be used interchangeably with the WAnT, especially in adolescent volleyball players, this did not imply that the latter test was not suitable for monitoring volleyball players. The WAnT has been used extensively to evaluate anaerobic profile in this sport [8,30,31,32]. On the other hand, jumping tests provided additional information about sport-specific qualities, such as the stretch-shortening cycle or the use of the arm-swing.

## 5. Conclusions

In summary, the impact of the Bosco test and the WAnT on muscle power varied, especially in the younger age group. Single vertical jumping tests had larger correlations with WAnT in adult rather than in adolescent volleyball players. These findings should be taken into account by volleyball coaches and fitness trainers during the assessment of short-term muscle power of their athletes.
